# The development and internal validation of a model to predict functional recovery after trauma

**DOI:** 10.1371/journal.pone.0213510

**Published:** 2019-03-14

**Authors:** Max W. de Graaf, Inge H. F. Reininga, Erik Heineman, Mostafa El Moumni

**Affiliations:** 1 Department of Trauma Surgery, University Medical Center Groningen, University of Groningen, Groningen, The Netherlands; 2 Department of Surgery, University Medical Center Groningen, University of Groningen, Groningen, The Netherlands; Cedars-Sinai Medical Center, UNITED STATES

## Abstract

**Objective:**

To develop and internally validate the PROgnosis of functional recovery after Trauma (PRO-Trauma) prediction model.

**Design:**

A prospective single-center longitudinal cohort study. Patients were assessed at 6 weeks and 12 months post-injury.

**Methods:**

Patients that presented at the emergency department with an acute traumatic injury, were prompted for participation. Patients that completed the assessments at 6 weeks and 12 months post injury were included. Exclusion criteria: age < 18, age > 65, pathologic fractures, injuries that resulted in severe neurologic deficits. The predicted outcome, *functional recovery*, was defined as a Short Musculoskeletal Function Assessment (SMFA-NL) Problems with Daily Activities (PDA) subscale ≤ 12.2 points at 12 months post-injury (Dutch population norm). Predictors were: gender, age, living with partner, number of chronic health conditions, SMFA-NL PDA score 6 weeks post-injury, ICU admission, length of stay in hospital, injury severity score, occurrence of complications and treatment type. All predictors were obtained before 6 weeks post-injury. Missing data were multiply imputed. Predictor variables were selected using backward stepwise multivariable logistic regression. Hosmer-Lemeshow tests were used to evaluate calibration. Bootstrap resampling was used to internally validate the final model.

**Results:**

A total of 246 patients were included, of which 104 (44%) showed functional recovery. The predictors in the final PRO-Trauma model were: living with partner, the number of chronic health conditions, SMFA-NL PDA subscale score at 6 weeks post-injury and length of stay in hospital. The apparent R^2^ was 0.33 [0.33;0.34], the c-statistic was 0.79 [0.79;0.80]. Hosmer-Lemeshow test indicated good calibration (p = 0.92). Optimism-corrected R^2^ was 0.28 [0.27;0.29] and the optimism-corrected Area Under the Curve was 0.77 [0.77;0.77].

**Conclusion:**

The PRO-Trauma prediction model can be used to obtain valid predictions of attaining functional recovery after trauma at 12 months post-injury. The PRO-Trauma prediction model showed acceptable calibration and discrimination.

## Background

In trauma surgery, clinical outcomes and treatment effects have traditionally been assessed with measures such as mortality rates, non-union rates, radiographic evaluation of bone healing, and clinician-based measurements such as range of motion. Unfortunately, these measures of outcome, have shown to correlate poorly with patients’ view of their *physical functioning* (see Box 1) [1,2]. In the past decades, the number of deaths due to trauma has declined [3]. As a consequence, the burden of trauma has largely shifted from fatal to non-fatal outcomes [4].

**Box 1. Definition of physical functioning**

**Physical functioning**: one’s ability to carry out activities that require physical actions, ranging from self-care (activities of daily living) to more complex activities that require a combination of skills, often within a social context [2].

Injury-related disability and recovery of physical functioning are complex problems that are influenced by a multitude of observable and unobservable factors. A limited number of studies have investigated which factors influence physical functioning after trauma. Variables that have consistently been associated with long-term physical functioning, are: age, gender, comorbidities, complications, length of stay, ICU admission and injury severity [1,5–8].

A gap in the present literature is that, despite multiple variables have been associated with physical functioning after trauma, they have never been unified to a model that can *predict* recovery of physical functioning after trauma. The need for an instrument that is capable of predicting patients’ chance on recovery of physical functioning (e.g. functional recovery) has been explicated previously [4,8].

Instruments that provide a probability of the occurrence of a future event, are important in clinical practice [9]. A prognostic model to predict functional recovery after trauma, enables clinicians to systematically identify patients that are at risk of developing a poor functional outcome in an early stage. In addition, patients may be informed earlier, with quantified estimates of their chance on functional recovery.

The aim of this study was to develop and internally validate the PROgnosis of functional recovery after Trauma (PRO-Trauma) model. A multivariable prognostic model, that can be used to estimate the individual chance on attaining functional recovery at 12 months post-injury, in patients with a broad range of acute traumatic injuries.

## Materials and methods

### Study design and source of data

The PRO-Trauma model was developed from the data of a prospective single center longitudinal cohort study that was used for the evaluation of the measurement properties of the Dutch Short Musculoskeletal Function Assessment (SMFA-NL) questionnaire [10]. In the cohort, patients filled in the SMFA-NL at 6 weeks post-injury and 12 months post-injury. Patients were enrolled from October 2012 until March 2016.

Patients, between 18 and 65 years of age, were prompted for participation when they had visited the emergency department the University Medical Center Groningen (a level 1 trauma center, The Netherlands) with an acute traumatic injury. Patients were included when they participated in both the 6 weeks and 12 months post-injury measurements. Exclusion criteria were: inability to read or write Dutch, severe traumatic brain injury, injuries that resulted in severe neurologic deficits, pathologic fractures and patients with severe psychiatric or cognitively impaired conditions, minor injuries that required treatment at the outpatient clinic for four weeks or less.

The study design has been reviewed by the Institutional Review Board, of the University Medical Center Groningen, and waived further need for approval (METc2012.104). Patients consented with participation in the study. The study was carried out in compliance with the principles outlined in the Declaration of Helsinki [11]. The model was developed and reported following the Transparent Reporting of a multivariable prediction model for Individual Prognosis or Diagnosis (TRIPOD) statement [12].

### Short Musculoskeletal Function Assessment

The SMFA-NL is a patient-reported outcome measure (PROM) that can be used to evaluate physical functioning at one moment, and as follow-up instrument to evaluate physical functioning over time [13]. The SMFA-NL consists of four subscales: Upper Extremity Dysfunction, Lower Extremity Dysfunction, Problems with Daily Activities and Mental and Emotional Problems [10]. Items are scored on a 5-point Likert scale. Subscale scores range from 0 to 100. A score of 0 represents best function. The SMFA-NL has demonstrated sufficient validity, reliability and responsiveness in traumatically injured patients [10]. The SMFA-NL was administered on-paper or electronically.

### Outcome

The predicted outcome was *functional recovery* at 12 months post-injury. *Functional recovery* was defined as an SMFA-NL Problems with Daily Activities subscale score ≤ 12.2 points (normative value) at 12 months post-injury. The SMFA-NL Problems with Daily Activities subscale has shown to correlate strongly with both generic health-related quality of life instruments, and instruments that evaluate upper and lower extremity function [10]. Therefore the Problems with Daily Activities subscale was considered a measure of general physical functioning. The Dutch population normative value of the Problems with Daily Activities subscale is 12.2 points, and was considered to represent a level of adequate physical functioning [14,15]. Patients that scored equal or better than the population norm, were considered to be functionally recovered.

### Predictors

The predictors were required to cover multiple facets that influence functional recovery. Candidate predictor variables were selected upon literature review followed by consensus of a panel that consisted of a trauma surgeon-epidemiologist, a human movement scientist-epidemiologist and a general physician. Both ‘patient-reported’ and ‘physician-reported’ predictors were selected. Predictor variables were all collected before 6 weeks post-injury.

Patient-reported predictors were: age at time of the injury, gender, living with partner, the number of chronic health conditions and the SMFA Problems with Daily Activities score at 6 weeks post injury. Units, source and additional information regarding candidate predictors are shown in S1 Table. The number of chronic health conditions were calculated from the presence of 12 common chronic health conditions (S1 Table) [16,17].

Physician-reported predictors were: treatment type (surgical or conservative treatment), presence of an injury or surgery-related complication, length of stay in hospital, Intensive Care Unit (ICU) admission, Injury Severity Score (S1 Table) [18]. Examples of injury or surgery-related complications were re-bleeding, wound infection, re-fracture, failure of osteosynthesis material.

### Sample size

The required sample size was derived from the number of predictors in the model. Previous studies have suggested that about 10 events (patients that recovered) are required per predictor variable, in order to avoid over-fitting [19,20]. We aimed to evaluate 10 predictor variables in the model, therefore at least 100 events were required.

### Missing data

Missing data was handled using multiple imputation [21]. Missingness at random was assumed. In order to impute with greatest accuracy, imputations were predicted by the outcome variable, all candidate predictors and auxiliary variables with a correlation of at least 0.4 with the imputed variable [22]. Continuous variables were imputed using predictive mean matching. Binary variables and categorical variables were imputed using logistic and polytomous regression, respectively [22]. The number of imputed datasets was guided by Von Hippel, using a coefficient-of-variation of 0.05 [23]. A total of 5 imputed datasets were generated, using 50 iterations. The accuracy and acceptability of the imputed data was evaluated with distribution plots and propensity plots [24,25].

### Model development and statistical analysis

#### Data preparation

The 6 weeks and 12 months post-injury SMFA-NL Problems with Daily Activities subscale scores were calculated. The outcome functional recovery was defined by dichotomizing the 12-months post-injury SMFA-NL Problems with Daily Activities subscale score by 12.2 points [14].

#### Selection of predictor variables

A multivariable logistic regression analysis was used to evaluate the relation of the predictors with the outcome functional recovery in each imputed dataset. All predictors were considered in the full model and were eliminated using backward stepwise selection. To decide which predictors should be kept in the final model, predictors were removed from the pooled model, using the Pooled Sampling Variance (D1) method [26,27]. A p-removal value of 0.157 was used to warrant inclusion and prevent omission of important predictors [9,12]. The final model was pooled using Rubin’s rules [28,29].

#### Calibration and apparent performance

Calibration was evaluated using Hosmer-Lemeshow tests in each of the imputed datasets. A median [Q_1_; Q_3_] p-value > 0.05 was considered evidence of good calibration [29]. In addition, calibration plots were constructed in each dataset. The apparent performance was examined using the median [Q_1_; Q_3_] Nagelkerke’s R^2^ and the c-statistic across the imputed datasets [29]. A c-statistic of 1 reflects perfect prediction, a value of 0.5 reflects no discrimination beyond random chance. Criteria for the c-statistic were: 0.7 ≤ c-statistic < 0.8 = ‘acceptable discrimination’, 0.8 ≤ c-statistic < 0.9 = ‘excellent discrimination’, c-statistic ≥ 0.9 = ‘outstanding discrimination’ [30].

#### Internal validation and adjusted performance measures

The developed model was internally validated using bootstrap resampling. In each dataset, 200 bootstrap samples were drawn. In each bootstrapped sample, the developed model was re-estimated using backward stepwise selection [31]. Optimism-corrected performance was calculated as: Optimism-corrected performance = apparent performance–optimisim [9]. Optimism-corrected coefficients and intercepts were calculated and were pooled using Rubin’s Rules [28,29]. The median [Q_1_; Q_3_] optimism-corrected Nagelkerke’s R^2^ and c-statistic were calculated [29].

A nomogram was constructed to facilitate easy calculation of risk scores and probabilities of reaching functional recovery. A clinical example was described in which the prediction model and nomogram were demonstrated to evaluate the chance on reaching functional recovery.

#### Sensitivity analysis

BMI and smoking status were collected after 12 months post-injury and could therefore not be included in the development of the model. It was hypothesized that BMI and smoking status 12 months post-injury were representative for BMI and smoking status at 6 weeks post-injury. In the sensitivity analysis, the selection of predictor variables was repeated, including BMI and smoking status. The selected predictors and apparent performance of the model were reported.

Statistical analysis was performed in R: A Language and Environment for Statistical Computing [32]. Model development was performed with R-package PSFMI; the internal validation and nomogram construction were performed using R-package RMS [33,34].

## Results

### Sample characteristics

A total of 246 patients participated in the study (138, 56%, men). In the original un-imputed dataset, a total of 104 (44%) patients showed functional recovery. Upper and lower extremity injuries were most prevalent (Table 1). The majority of the patients received surgical treatment (52%) and the mean injury severity score was 6.8. The average SMFA-NL Problems with Daily Activities score at 6 weeks post-injury was 50.3 points (Table 1).

**Table 1:** General characteristics.

		N (%)	Missing (%)	Imputed N(%) Mean of 5 datasets
**Predicted outcome**				
Functional Recovery		104 (44)	10 (4)	108 (44)
**Patient-reported predictors**				
Gender (n = 246)			0	n.a.
	Male	138 (56)		
	Female	108 (44)		
Age		48 (13.0)	0	n.a.
Living with partner			13 (5)	
	Single	81 (35)		85 (35)
	With partner	152 (65)		162 (65)
Educational level			15 (6)	
	Elementary school	4 (2)		5 (2)
	High school	73 (32)		77 (32)
	College	66 (29)		69 (28)
	Bachelors degree or higher	88 (38)		95 (39)
Number of chronic health conditions				
	None			
	One			
	Two or more			
SMFA-NL PDA score 6w post-injury*		50.3 (25.3)	9 (4)	50.1 (25.1)
**Physician-reported predictors**				
Injuries			0	n.a.
	Head and neck	35		
	Face	18		
	Thorax	55		
	Abdomen	13		
	Spine	83		
	Upper extremity	153		
	Lower extremity and pelvic bones	181		
	Skin^2^/other	60		
Injury Severity Score^1^		4 (6.2)	0	n.a.
Treatment			0	n.a.
	Conservative treatment	117 (48)		
	Surgery	129 (52)		
Injury or surgery related complication		33 (13)	0	n.a.
Length of stay in hospital^1^		6 (9.4)	2	6 (9.4)
Intensive Care Unit admission		17 (7)	0	n.a.
**Sensitivity Analysis**				
	Smoking status	29 (23)	118 (47)	61 (25)
	BMI^1^	25.7 (4.9)	138 (56)	25.9 (5.9)

^1^Presented as mean (SD)

^2^Superficial injuries (abrasion, contusion, lacerations regardless of anatomical region. N.a.: not applicable since: no missing values

* SMFA-NL Problems with Daily Activities at 6 weeks post-injury

Percentage of missingness per variable is shown in Table 1. Smoking status and BMI, assessed at 12 months post-injury, were missing most. The imputed data overlapped the distribution of the observed data, indicating realistic imputations and were accepted for further analysis (S1 Fig). The characteristics of the 5 complete datasets were shown in Table 1.

### Model development

All patient-reported and physician-reported predictors were used in the full model. The final PRO-Trauma model consisted of four predictors: the SMFA-NL Problems with Daily Activities scale 6 weeks post-injury, living with partner, number of chronic health conditions, length of stay in hospital. The regression coefficients, standard errors and odds ratios of the final model were shown in Table 2.

**Table 2:** Regression coefficients of the apparent and internally validated PRO-Trauma model.

		Functional Recovery				
		Development				Internal validation
		ß-coefficient	Standard error	Odds ratio	p-value	Optimism-corrected ß-coefficient
Intercept		-1.826	0.400	0.16	n.a.	-1.546
SMFA-NL PDA 6w post-injury		0.030	0.007	1.03	<0.001	0.026
Living with partner		-0.652	0.340	0.52	0.06	-0.561
Number of chronic health conditions					<0.001	
	None	0	n.a.	1.0		
	One	1.331	0.379	3.78		1.146
	Two or more	1.677	0.460	5.34		1.442
Length of stay in hospital		0.059	0.023	1.06	0.01	0.051

PRO-Trauma: PROgnosis of functional recovery after Tauma, ß-coefficient: unstandardized regression coefficient. SMFA-NL PDA 6w: The Short Musculoskeletal Function Assessment Problems with Daily Activities subscale score at 6 weeks post-injury. N.a.: not applicable.

**Fig 1:**
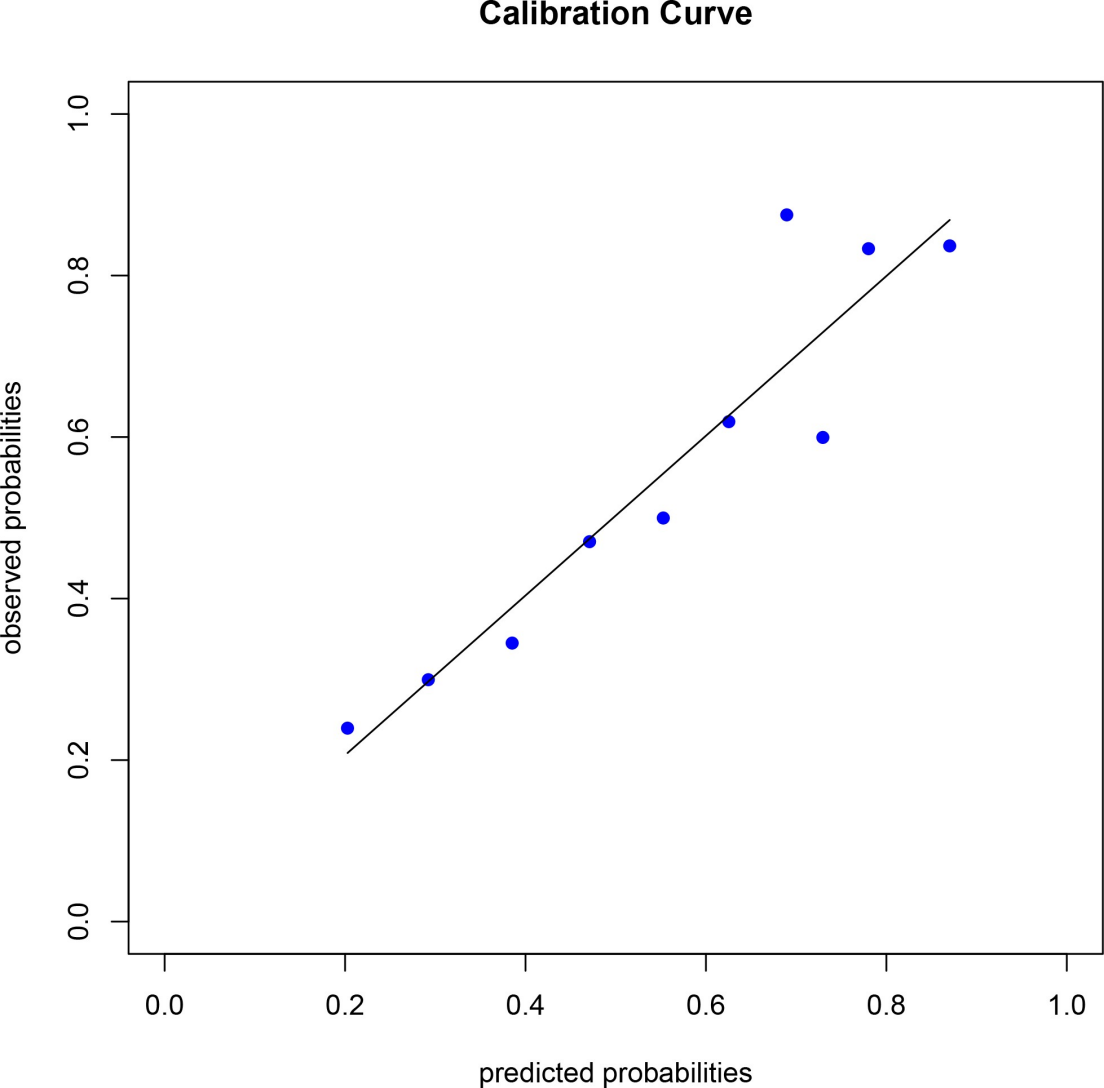
Calibration curve of the final PROgnosis of functional recovery after Trauma model in dataset 4. The solid black line indicated perfect calibration, the blue dots indicated the actual model calibration. Dataset 4 was randomly chosen. Other calibration plots are available as supporting information.

The median Nagelkerke’s R^2^ was 0.33 [0.33; 0.35]. The median c-statistic was 0.79 [0.79; 0.80], indicating acceptable discrimination. The Hosmer-Lemeshow test showed a median p-value of 0.93 [0.90; 0.94], indicating good calibration. This was supported by the calibration curve of the PRO-Trauma model in each imputed dataset (Fig 1 and S2–S5 Figs).

### Internal validation and optimism-corrected performance measures

The median optimism-corrected Nagelkerke’s R^2^ was 0.28 [0.27; 0.29]. The median optimism-corrected c-statistic was 0.77 [0.77; 0.77], indicating acceptable discrimination. The optimism-corrected regression coefficients and intercept are shown in the last column of Table 2 and Box 2.

**Box 2. Individual risk score and probabilities**

Risk Score = 1.546

+ 0.026 * SMFA-NL Problems with Daily Activities score 6weeks post injury *

+ 0.051 * Length of stay in hospital ^†^

- 0.561 [if living with partner] ^‡^

+ 1.146 [if ONE chronic health condition present] ^§^

+ 1.442 [if TWO OR MORE chronic health conditions present] ^§^

$${Probability}_{Functional\;Recovery}=\frac{1}{1+e^{Risk\;Score}}$$

A probability of 1 represents highest chance on functional recovery. * Score in points, ranging from 0 to 100. 100 is represents best function. † Length of stay in hospital in days. ‡ If not living with partner, no points should be given. § If no chronic health conditions present, no points should be given. Chronic health conditions are: migraine, hypertension, either asthma or COPD, severe back conditions, severe gut-related disease, osteoarthritis, rheumatoid arthritis, diabetes mellitus, stroke, myocardial infarction, severe non-infarct conditions, malignant disease.

### Application of the PRO-Trauma model

**Fig 2:**
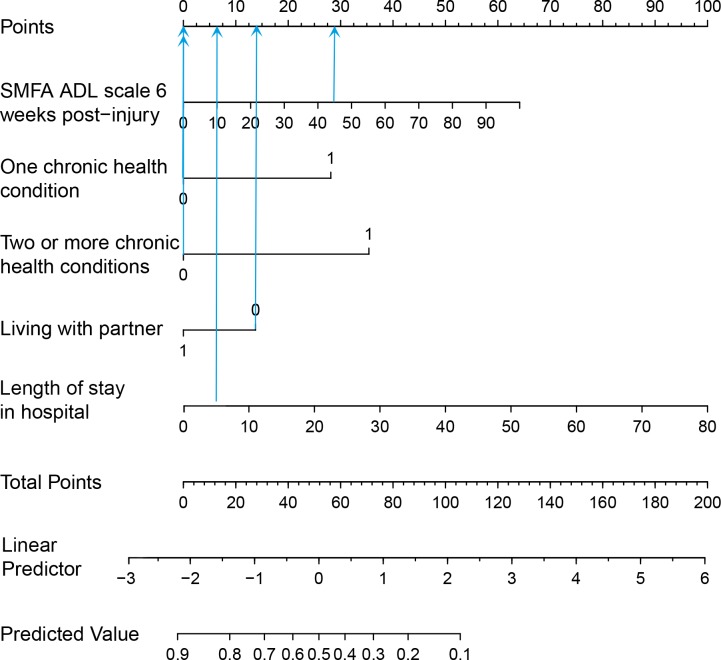
Nomogram of the PROgnosis of functional recovery after Trauma model. The arrows in the nomogram represent the designated points from the clinical example. With the nomogram the probability of attaining functional recovery can be estimated as follows: 1) the value of each predictor can be designated points by drawing a straight line from the predictor up to the ‘Points’ line. For example the values of the clinical example are: no chronic health conditions: 0 points; living with partner: 13 points; SMFA-NL ADL: 29 points; length of stay in hospital: 6 points. 2) The rewarded points can be summed to a total (Total Points line). Total of the clinical example: 48 points. 3) The chance on attaining functional recovery can be obtained by drawing a straight line from the Total Points line, down to the ‘Predicted Value’ line. In the clinical example the predicted probability is 0.54 of being functionally recovered after one year.

Individual predictions can be obtained by calculating a risk score (e.g. linear predictor score), shown in Box 2. The calculated risk score can be entered in the formula shown in Box 2, which results the probability of functional recovery at 12 months post-injury. A nomogram was generated to facilitate easy application in an outpatient clinic or in a bed-side environment (Fig 2).

Clinical example: A 25 year-old man, was admitted due to motor vehicle injury. Had no chronic health conditions and lived without a partner. The patient was diagnosed with a left-sided femoral shaft fracture, for which he was treated with an antegrade femoral nail. The patient was dismissed after 5 days. At 6 weeks post-injury the SMFA-NL Problems with Daily Activities subscale score was 44 points. The Risk Score (linear predictor) was -0.147, which corresponds to a 54% chance on functional recovery after 1 year. In the nomogram, each variable was rewarded points; the variables summed to 48 points in total, corresponding with a probability on functional recovery of about 54% (Fig 2).

### Sensitivity analysis

The selected model consisted of the SMFA-NL Problems with Daily Activities scale 6 weeks post-injury, BMI (ß = -0.07, p = 0.06), smoking status (ß = 0.88, p = 0.10), living with partner, number of chronic health conditions, length of stay in hospital. The median Hosmer-Lemeshow test p-value was 0.81 [0.63;0.83], indicating good calibration. The median Nagelkerke’s R^2^ was 0.41 [0.40; 0.42] and the median C-statistic was 0.83 [0.82;0.83].

## Discussion

### Principal findings of the study

The PRO-Trauma prediction model provided estimates of the overall chance on being functionally recovered at 12 months post-injury. The model consisted of four variables: SMFA-NL Problems with Daily Activities, length of stay in hospital, living with a partner and the number of chronic health conditions. The PRO-Trauma prediction model showed acceptable calibration and discrimination. Predictions can be made in an early stage of the recovery trajectory: at 6 weeks post-injury.

### Interpretation of the findings

The predictors of the final model, were clinically relevant and sustained by previous literature [1,5–8]. The number of chronic health conditions was the strongest predictor. In Western populations, the burden of chronic health conditions is increasing and causes an important reduction in quality of life [35]. The present study showed that chronic health conditions also have a profound impact on the recovery of physical functioning. Consequently, functional limitations after trauma can lead to physical inactivity, which may worsen chronic health conditions and quality of life [36]. These findings encourage a proper monitoring of chronic health conditions after trauma, such that recovery is minimally affected by chronic disease and vice versa.

PROMs such as the SMFA-NL, are mostly used as an end-point, to evaluate functional outcome. However, PROMS such as the SMFA-NL, may also serve as a quality-control instrument for benchmarking performance of healthcare institutions, or as clinical follow-up instrument that measures change in physical functioning over time [10,37]. The present study showed that physical functioning shortly evaluated after the injury, is a predictor of long-term chance on reaching functional recovery. Therefore, this study showed that clinical follow-up instruments may be used to predict future outcome. In the literature, the predictive capacity of follow-up instruments is not frequently investigated. However, we think it provides an important opportunity to gain insight into long-term outcome after trauma, and should be studied more thoroughly.

Patients without a partner were at risk of not reaching functional recovery. We think this may reflect the strength of patients’ social network and available social support. Partners may provide support in coping, as well as providing aid to overcome a patient’s own activity limitations and participation restrictions. Having a partner and a strong social network have been associated with better functional outcome and higher return to work rates [38,39]. Although more research is needed to understand the role and possible use of patients’ social networks on (the improvement of) functional outcome after trauma, the importance of a social network with respect to functional recovery should not be underestimated [38,39].

Length of stay in hospital was an independent predictor of functional recovery. The injury severity score (ISS), was not. The ISS is an established score to rate the overall severity of injury in patients with one or more injuries. The ISS takes into account anatomic injury region, injury type and the number of injuries. However, the ISS did not provide additional significant prognostic value and was removed from the model in the backward-stepwise selection procedure. This may be related to the construct that the ISS intends to measure. Namely, the ISS was developed to predict trauma related mortality, and not morbidity or functional outcome [8,18]. Though several studies reported the association of ISS with functional outcome, the majority reported that the ISS was not associated with functional outcome [1,4,6–8,40]. Further research is required to develop a valid classification that relates injury severity to the impact on physical functioning.

Smoking and BMI were both identified as independent predictors of functional recovery in the sensitivity analysis. The negative effects of smoking and sub-optimal BMI on general health are widely known. These negative effects also appeared to apply to the chance of being functionally recovered after 12 months. Both parameters could not be included in the model, since both were assessed at 12 months post-injury. However, it is likely that the c-statistic of the PRO-Trauma model would have been higher, if these variables could have been included.

### Comparison with existing prognostic models

In trauma surgery, evaluation of treatment outcome, traditionally focused on mortality. As a consequence, multiple prognostic models were developed to predict mortality after trauma, including TRISS, ISS and the Revised Trauma Score [41]. However, since the vast majority of all trauma patients survive, there is a need for a model that is able to predict functional outcome. To the best of our knowledge, a model that predicts functional recovery after trauma has never been reported. The PRO-Trauma prediction model is difficult to compare to the mortality-related prediction models, since it is different in many aspects. Foremost, our model predicts functional recovery, instead of mortality. Furthermore, the PRO-Trauma prediction model used a holistic approach, using predictors that related to various different aspects of functional recovery, instead of including only anatomical, physiological or patient characteristics. Despite the scarcity of prognostic models that predict outcome after trauma, there appears to be increasing attention for the development of such models. Recently, De Jongh et al. proposed a study protocol of a longitudinal cohort study, in which one of the objectives is to develop and validate a prognostic model for predicting functional outcome after trauma [42]. However, results have not yet been reported.

In a related clinical field, two prognostic models have been developed that predict clinical outcome in patients that sustained traumatic brain injury. Steyerberg et al. developed a model using physiological, biochemical and radiographic predictors, on the Glasgow Outcome Scale (GOS) at 6 months post-injury [43]. The AUC (which is identical to the c-statistic) ranged from 0.66 to 0.84. Hukkelhoven et al. developed a similar model using the GOS and reported AUC values ranging from 0.78 to 0.80 [44]. The PRO-Trauma model showed a comparable discriminative performance compared to the models in patients with traumatic brain injury.

The PRO-Trauma prediction model was developed for patients of the working-age without traumatic brain injury or severe neurologic deficits. Traumatic brain injury and severe neurologic deficits due to trauma have vastly different recovery patterns in terms of physical functioning compared to patients with musculoskeletal injuries. Both the SMFA questionnaire and PRO-Trauma model were not developed and validated for patients with such conditions. Without a specific validation procedure in these patients, the model may not be used in patients with such injuries. In addition the PRO-Trauma prediction model cannot be directly applied to geriatric trauma patients. These patients were not part of the study sample. There is accumulating evidence that injury types, severity and recovery patterns after trauma in elderly follows distinct patterns compared to younger patients [45,46]. Elderly often have diminished pre-injury functional capacities and frequently have suboptimal physiological and cognitive reserves compared to patients of the working age. We actively encourage development of similar prognostic models for these patient groups.

### Clinical application

Early prediction of functional recovery, allows clinicians to systematically identify patients that are at risk of developing a poor functional outcome. In addition, clinicians may inform patients with a quantified estimate of their chance on functional recovery, which may aid in shared decision making. Though, it should be noted that clinical prediction models are an augmentation to clinical appreciation of patients, and should not replace clinical judgment of patients that sustained an injury. Clinicians may prefer the nomogram for a quick and comprehensive assessment. Exact probabilities may be calculated by hand or using the provided Excel sheet (electronic supplement).

The PRO-Trauma prediction model may be useful in clinical research. Being able to identify which patients are at risk of a poor outcome, may have relevant consequences for the design and analysis of clinical trials. For example, patients at risk of poor functional outcome may be specifically included in an RCT, or risk scores may be used to adjust for treatment effects [43].

### Strengths and limitations

Fitting a large number of predictors carries the risk of overfitting, thereby making the findings less robust [19,20]. The sample size of the present study resulted in an event per variable (EPV) ratio of 10:1, thereby minimizing the risk of including irrelevant predictors to the model.

Missing data can seriously decrease the effective sample size and statistical power in regression models, even with a limited number of missing values [22]. In the present study, multiple imputation was used to handle missing values. Multiple imputation preserves sample size and statistical power with unbiased estimates and standard errors and has been advocated as preferred approach of handling missing data [47].

The validity and generalizability of clinical prediction models should be evaluated thoroughly before they can be used in clinical practice and decision making. One of the limitations of the present study was that only internal validity of the model was assessed. Internal validity is especially useful to evaluate the stability of the selected predictors and to appraise the quality of the predictions [48]. However, differences in healthcare systems and case-mix may influence the performance of prediction models. An additional external validation procedure, with data from a different hospital, is required to evaluate the generalizability and performance of the PRO-Trauma model.

Normative data of the SMFA-NL were used as a reference to indicate functional recovery [14]. General population norms can be considered to indicate a relatively high level of physical functioning. Lower thresholds could also have been used, such as a score of 0.5 SD under the population norm, or a score within one minimal important change (MIC) value of the population norm [15]. However, in the present study, the population norm value was chosen, so that patients at risk of a less favorable outcome would not be missed.

Even though the ISS was shown not to be a relevant predictor in the model, we think that the model does account for injury characteristics and injury severity, however not with traditional variables that directly reflect injury type or anatomic region. We think that the 6-weeks post-injury SMFA Problems with Daily Activities (PDA) subscale score and the Length of Hospital Stay predictors are reflective of injury severity and injury type. Patients with more severe injuries are likely to report a worse SMFA PDA score 6 weeks post-injury, which predicts a lower chance on functional recovery. In addition, more severely injured patients, are likely to have a longer length of hospital stay, which both predict a lower chance on functional recovery. Specification of injury type and anatomic region (for example by using the abbreviated injury scale as predictors) may improve the accuracy of the model, but would require creation of many predictor variables and/or categories. In the present study this was not feasible. Due to the sample size, additional variables could not be added to the model since this would violate the EPV ratio of 10:1 which was used to avoid overfitting and false-positive predictor selection. A secondary drawback of adding many injury type and region-specific variables is that it would complicate the model, which would negatively impact clinical usability. In future studies additional injury-specific variables may be evaluated to expand the PRO-Trauma prediction model, as well as other variables that may be predictive of functional recovery that were not available in the present study. Such predictors may be additional chronic health conditions such as kidney disease, radiographic signs such as (early) fracture consolidation, psychological factors, health literacy or a wider analysis of patients’ social support [39,49–52].

### Recommendations for future research

Prognosis of physical functioning after trauma is a complex context that is important to patients, health-care professionals and decision makers. The present study may be regarded as an initial exploring step in the direction of the development of prognostic models that predict outcome after trauma, rather than mortality. The external validation of the PRO-Trauma prediction model is required to evaluate the performance of the model in other populations and may be an important step towards clinical adoption. In addition, the PRO-Trauma prediction model may be expanded with additional predictors of functional recovery that improve the accuracy of the model. Though multiple longitudinal cohort studies have evaluated well-known predictors, a better understanding of factors that determine functional outcome after trauma is needed [1]. Due to the complexity of functional outcome after trauma, discovery of novel predictors and their relation to functional outcome after trauma is essential.

## Conclusion

In conclusion, a prognostic model that predicts functional recovery at 12 months post-injury is now available. The PRO-Trauma prediction model showed acceptable calibration and discrimination. Physical functioning shortly assessed after the injury, was predictive for functional recovery. The PRO-Trauma prediction model may be useful for quantifying the chance of reaching functional recovery at 12 months post-injury, identifying and informing patients that are at risk of developing a poor outcome and for adjusting treatment effects in clinical trials.

## Supporting information

S1 Fig Analysis of the imputed data: Imputation response probabilities. Original data = 0; Imputed datasets are 1 to 5. Blue circles: original observed data. Red dots: imputed data. The imputed data are within the observed data, indicating realistic imputations in each dataset.
(TIF)

S2 Fig Calibration plot of dataset 1. (TIF)

S3 Fig Calibration plot of dataset 2. (TIF)

S4 Fig Calibration plot of dataset 3. (TIF)

S5 Fig Calibration plot of dataset 5. (TIF)

S6 Fig Blank nomogram of the PROgnosis of functional recovery after Trauma model. This nomogram may be used and re-printed for use in clinical settings.
(TIF)

S1 Table Predictors considered in the model. (DOCX)
