# Supplementary figures and images for: The development and internal validation of a model to predict functional recovery after trauma
Source: PLoS One. 2019 Mar 14;14(3):e0213510. doi: 10.1371/journal.pone.0213510 (PMC6417777; doi:10.1371/journal.pone.0213510)

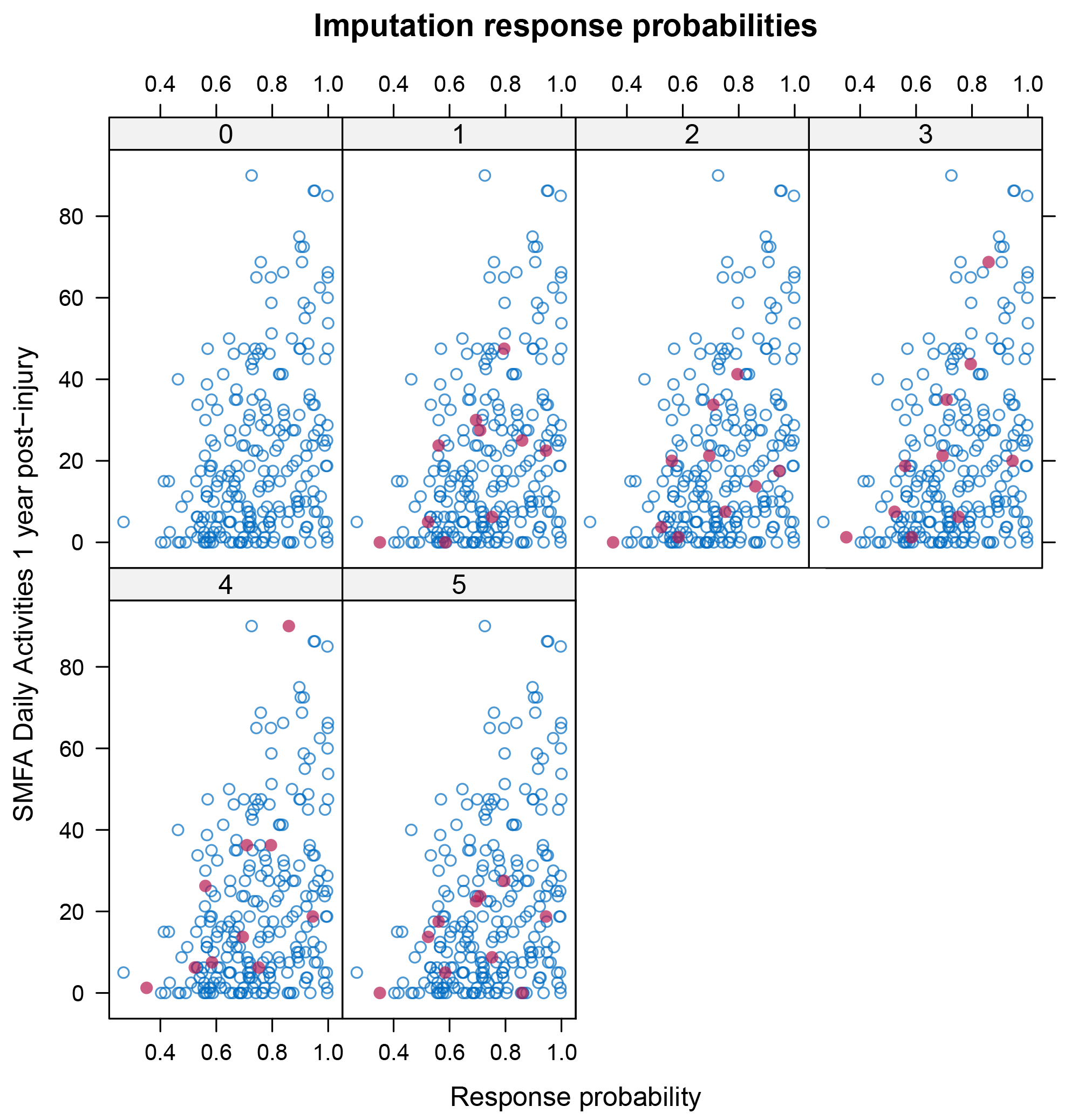

Supplement: S1 Fig — Original data = 0; Imputed datasets are 1 to 5. Blue circles: original observed data. Red dots: imputed data. The imputed data are within the observed data, indicating realistic imputations in each dataset. (TIF) [file pone.0213510.s001.tif]

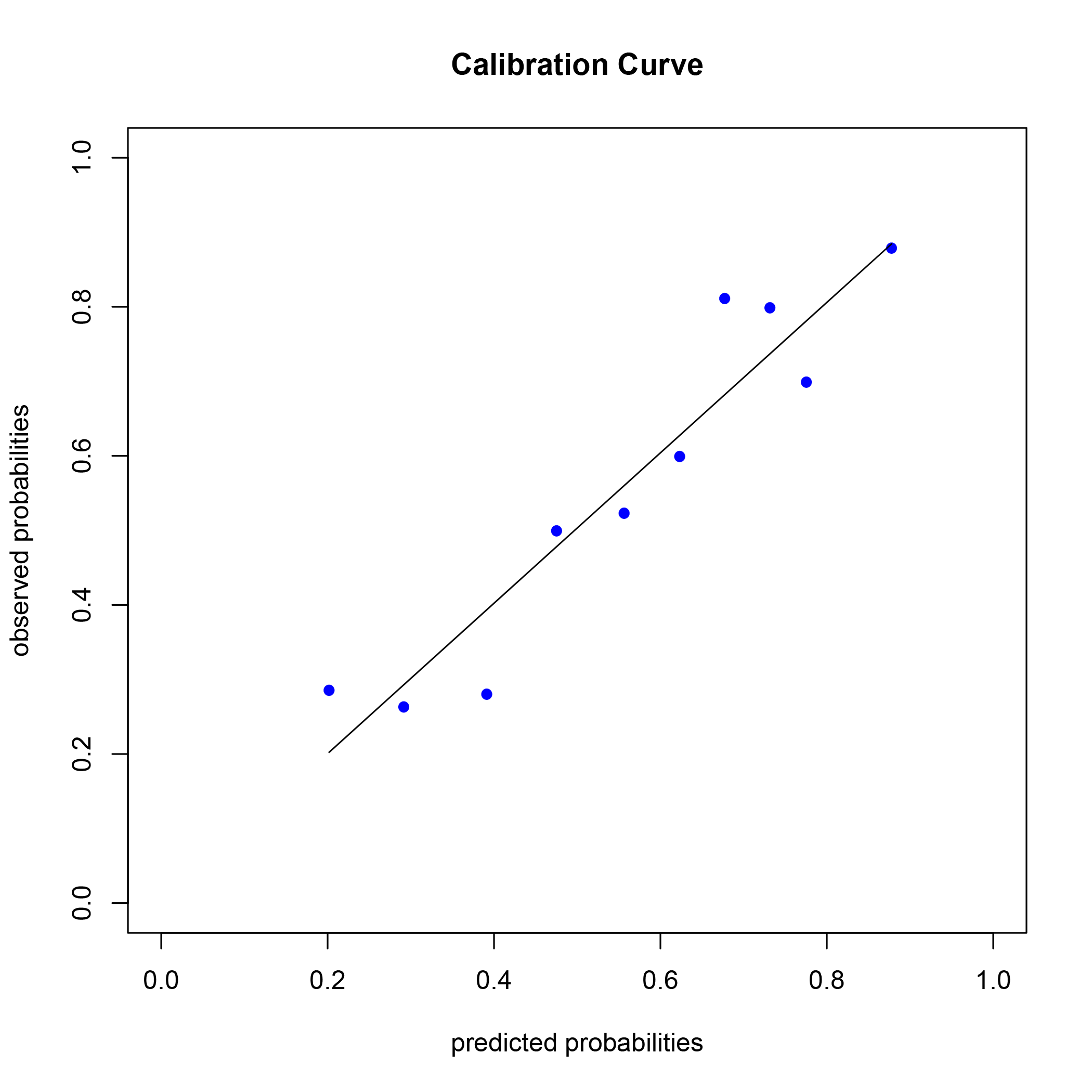

Supplement: S2 Fig — (TIF) [file pone.0213510.s002.tif]

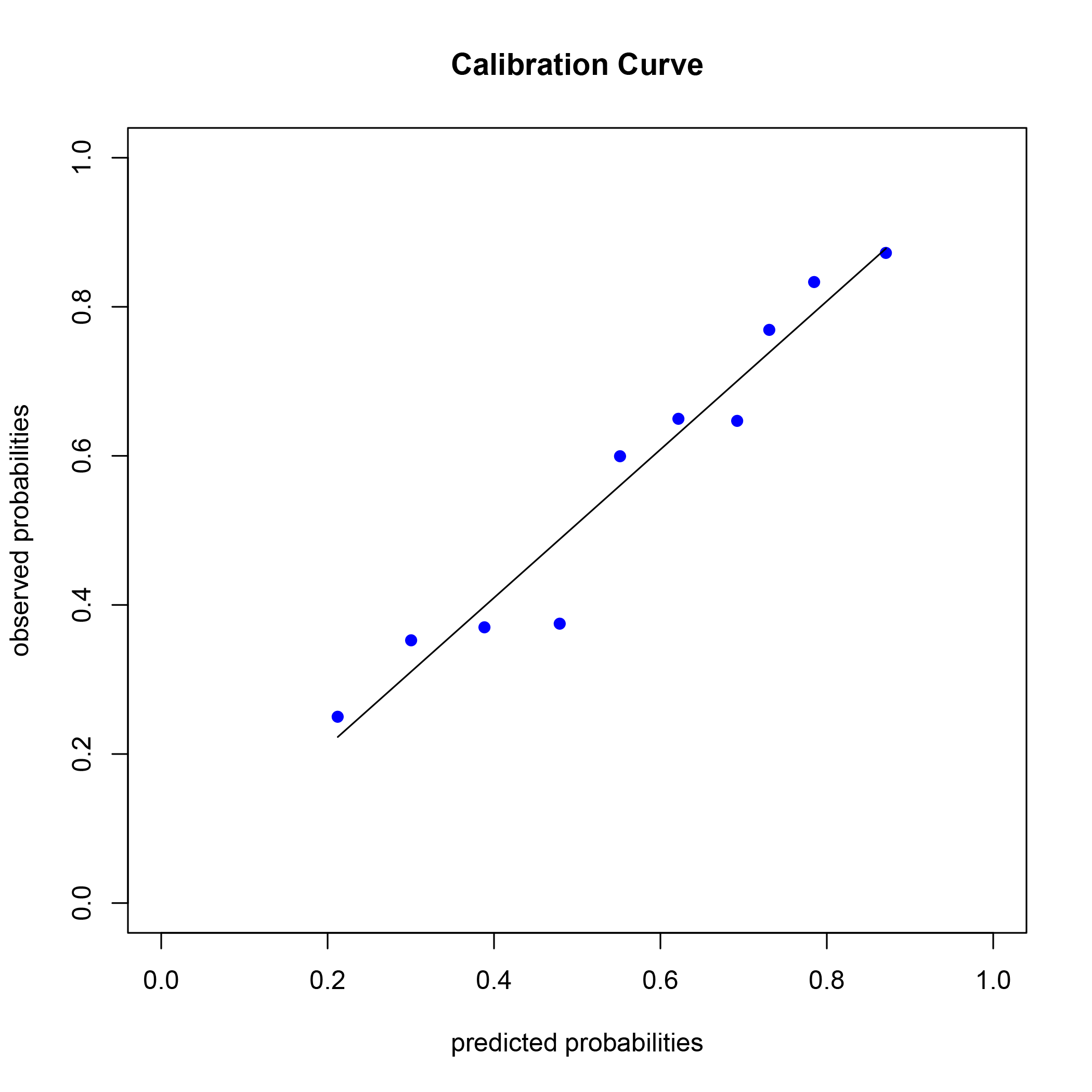

Supplement: S3 Fig — (TIF) [file pone.0213510.s003.tif]

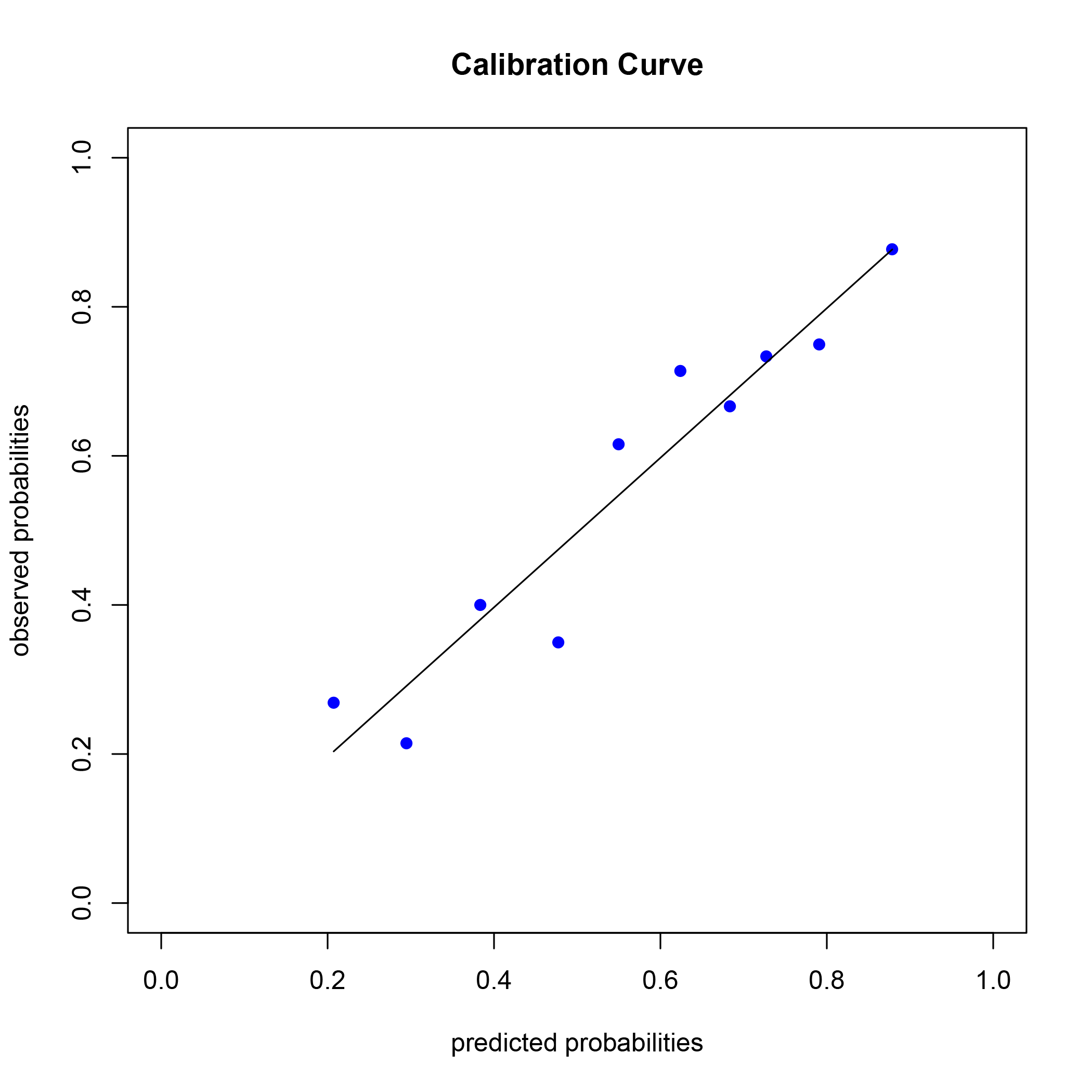

Supplement: S4 Fig — (TIF) [file pone.0213510.s004.tif]

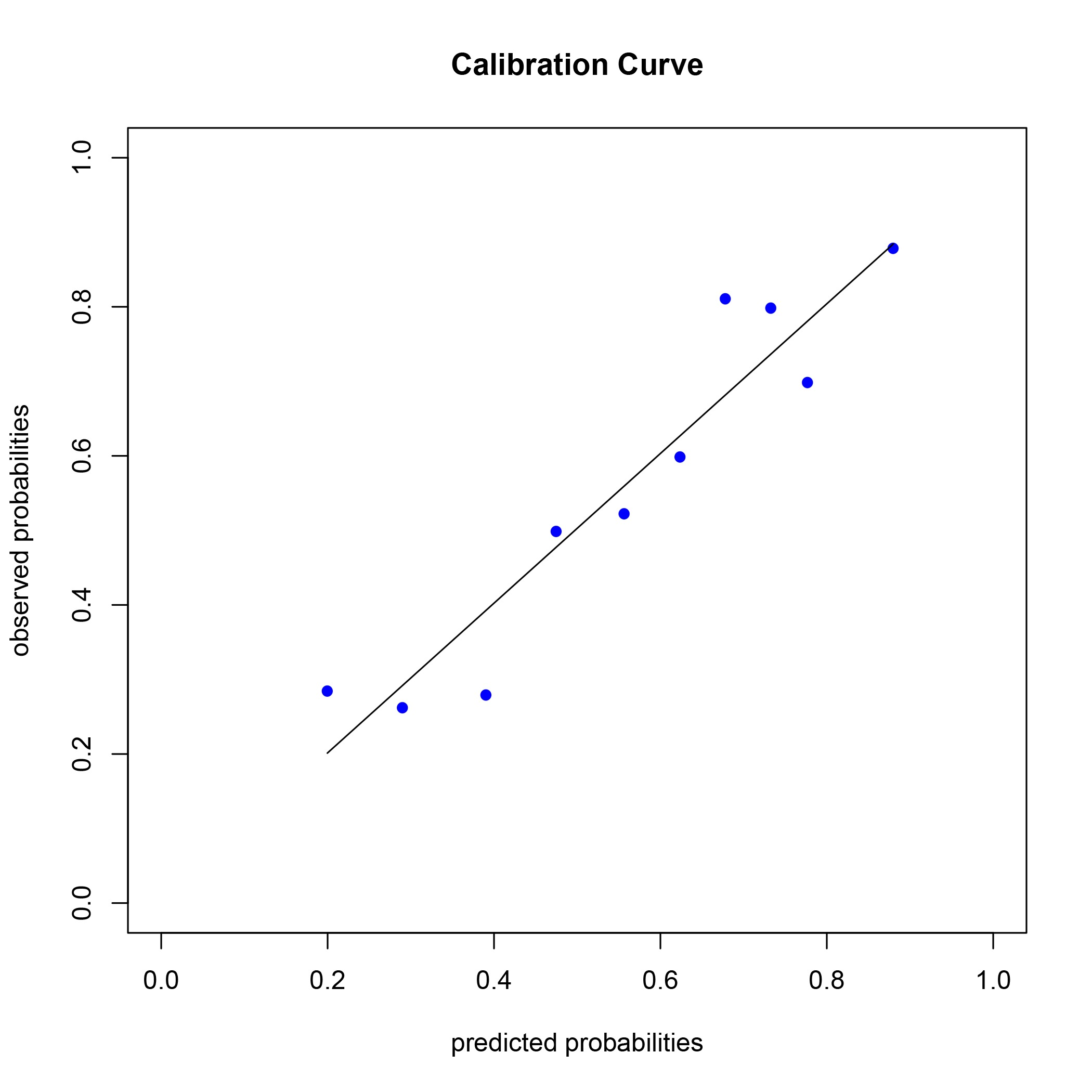

Supplement: S5 Fig — (TIF) [file pone.0213510.s005.tif]

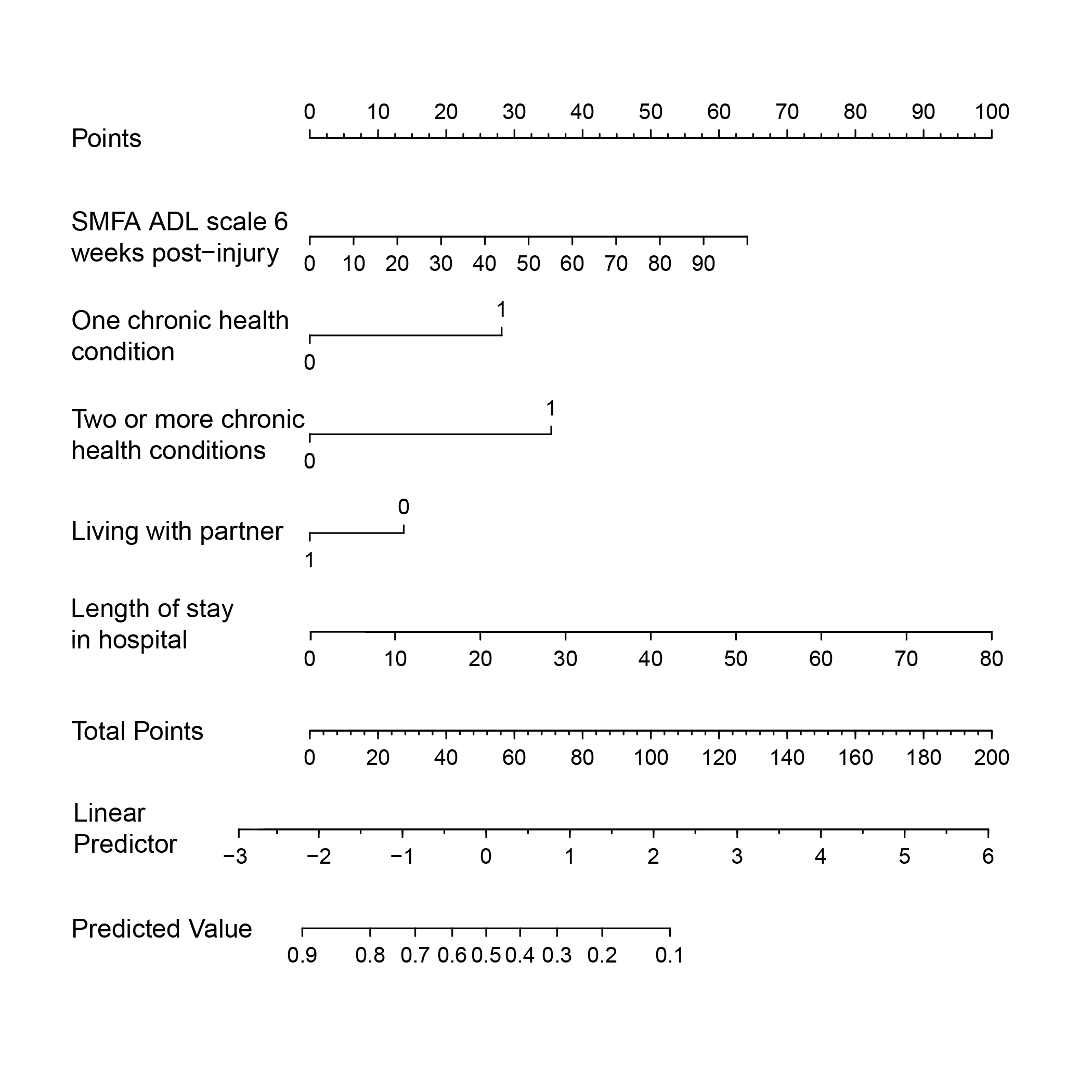

Supplement: S6 Fig — This nomogram may be used and re-printed for use in clinical settings. (TIF) [file pone.0213510.s006.tif]
