# Supplementary material for: The development and internal validation of a model to predict functional recovery after trauma
Source: PLoS One. 2019 Mar 14;14(3):e0213510. doi: 10.1371/journal.pone.0213510 (PMC6417777; doi:10.1371/journal.pone.0213510)
Supplement: S1 Table — (DOCX) [file pone.0213510.s007.docx]

|  | | | | |
| --- | --- | --- | --- | --- |
| Predictors | | Units or categories | Source | Additional information |
| **Patient-reported predictors** | |  |  |  |
|  | Age at time of the injury | years | Patient reported |  |
|  | Gender | Male/female | Patient reported |  |
|  | Living with partner | Alone/with partner | Patient reported |  |
|  | Number of chronic health conditions | 0, 1, 2 or more | Patient reported | Migraine, hypertension, asthma or COPD, severe back conditions, severe gut-related disease, osteoarthritis, rheumatoid arthritis, diabetes mellitus, stroke, myocardial infarction, severe non-infarct conditions, malignant disease |
|  | SMFA-NL Problems with Daily Activities score at 6 weeks post injury | 0-100 | Patient reported |  |
| **Physician-reported predictors** | |  |  |  |
|  | Treatment type | Surgery/conservative | Individual patient records | Surgery was defined as: patient received surgery for at least one injury. |
|  | Presence of an injury or surgery related complication | Yes/no | Institutional complication registry, patient records | Examples of injury or surgery-related complications were re-bleeding, wound infection, re-fracture, failure of osteosynthesis material |
|  | Length of Stay in Hospital | Days | Individual patient records |  |
|  | Intensive Care Unit admission | Yes/no | Individual patient records |  |
|  | Injury Severity Score | 0-75 | Trauma registry, individual patient records | Scored using AIS 2005, update 2008. |
| **Sensitivity Analysis** | |  |  |  |
|  | Smoking status (Daily smoking tobacco or e-cigarettes) | Yes/no | Patient reported | Collected 12 months post-injury. |
|  | Body Mass Index | Kg/m² | Patient reported | Mass/length². Collected 12 months post-injury |
|  | | | | |

**S1 Table: Predictors considered in the model**

SMFA-NL: Short Musculoskeletal Function Assessment, AIS: Abbreviated Injury Scale.
